# The potential of Fas ligand (apoptosis-inducing molecule) as an unconventional therapeutic target in type 1 diabetes

**DOI:** 10.3389/fimmu.2012.00196

**Published:** 2012-07-12

**Authors:** Abdel Rahim A.R Hamad, Kristin Arcara, Sophia Uddin, Thomas Donner

**Affiliations:** ^1^Department of Pathology, Johns Hopkins University School of Medicine,Baltimore,MD, USA; ^2^Department of Pediatrics, Johns Hopkins University School of Medicine,Baltimore, MD, USA; ^3^Department of Medicine, Johns Hopkins University School of Medicine,Baltimore, MD, USA

**Keywords:** autoimmune diabetes, Fas pathway, immunotherapy, immunosuppression, apoptosis, lymphoproliferative disorders

## Abstract

The development of type 1 diabetes (T1D) is driven by autoreactive T cells that attack and destroy the insulin-producing β-cells in pancreatic islets, forcing patients to take multiple daily insulin injections. Insulin therapy, however, is not a cure and diabetic patients often develop serious long-term microvascular and cardiovascular complications. Therefore, intensive efforts are being directed toward developing safe immunotherapy for the disease that does not impair host defense and preserves β-cells, leading to better glycemic control than exogenous insulin therapy. Engineering therapies that differentially cripple or tolerate autoreactive diabetogenic T cells while sparing protective T cells necessary for maintaining a competent immune system has proven challenging. Instead, recent efforts have focused on modulating or resetting the immune system through global but transient deletion of T cells or B cells using anti-CD3 or anti-CD20 mAb, respectively. However, phase III clinical trials have shown promising but modest efficacy so far with these approaches. Therefore, there is a need to identify novel biological targets that do not fit the classic properties of being involved in adaptive immune cell activation. In this prospective, we provide preclinical evidence that targeting Fas ligand (FasL) may provide a unique opportunity to prevent or cure T1D and perhaps other organ-specific autoimmune diseases without causing immune suppression. Unlike conventional targets that are involved in T and B lymphocyte activation (such as CD3 and CD20, respectively), FasL is an apoptosis-inducing surface molecule that triggers cell death by binding to Fas (also known as CD95 Apo-1). Therefore, targeting FasL is not expected to cause immune suppression, the Achilles Heel of conventional approaches. We will discuss the hypothesis that targeting FasL has unique benefits that are not offered by current immunomodulatory approaches.

## INTRODUCTION

Autoimmune diabetes, also known as type 1 diabetes (T1D), is a common chronic disease that strikes predominantly in childhood, adolescence or early adulthood and persists lifelong (Bluestone et al., 2010). It is clinically characterized by hyperglycemia due to the destruction of insulin-producing β-cells by diabetogenic T cells. Prior to the use of insulin for the management of T1D in 1922, T1D was invariably a fatal disease (Joslin, 1924, 1936). Since then, significant progress has been made in the development of long and short acting insulin analog regimens, home glucometers, continuous glucose sensors, and insulin pumps. Despite these advances, tight glycemic control remains an elusive and overwhelming challenge, requiring constant attention to blood glucose levels and carbohydrate intake (Patterson et al., 2009; Dahlquist et al., 2011; Harjutsalo et al., 2011). Patients continue to suffer from long-term diabetes complications including cardiovascular disease, chronic kidney disease, retinopathy which can progress to blindness, and peripheral neuropathy (Bluestone et al., 2010). Patients are also faced with the potential for life threatening episodes of diabetic ketoacidosis or hypoglycemia. T1D incidence has been steadily rising in young children for unclear reasons (Patterson et al., 2009; Dahlquist et al., 2011; Harjutsalo et al., 2011). Therefore, the need to develop a cure or preventive therapy for T1D is great. While a cure is defined as the lack of the need for exogenous insulin, developing a therapy that simply decreases the need for intensive insulin management and improve glycemic control would be transformative. Patients intensively treated with insulin in the Diabetes Control and Complications Trial (DCCT), who had higher baseline C-peptide concentrations (≥0.20 pmol/ml) indicating greater endogenous insulin secretion, on follow-up, had lower hemoglobin A1c levels, and a reduced risk for developing diabetic complications and severe hypoglycemia. Interventions which preserve endogenous insulin secretion may therefore improve control and prevent complications in patients with T1D (The Diabetes Control and Complications Trial Research Group, 1998).

## THE CHALLENGE TO DEVELOPING EFFECTIVE IMMUNOTHERAPY FOR T1D

**FIGURE 1:**
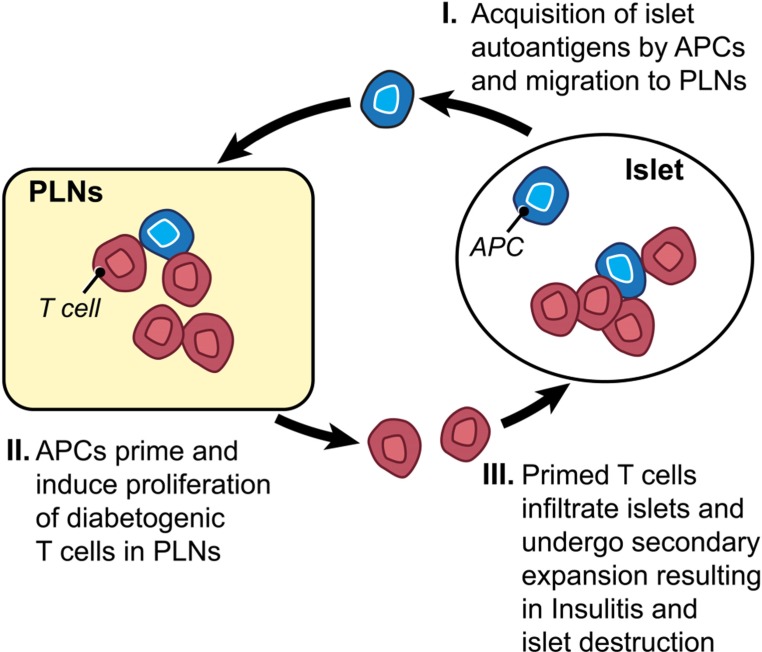
**Key events in T1D development.** (I) The process starts by acquisition and delivery of islet autoantigens including insulin and GAD65 proteins by antigen presenting cells (APCs) to the draining, pancreatic lymph nodes (PLNs). (II) Diabetogenic T cells encounter their cognate autoantigens presented by APCs in PLNs leading to their priming, proliferation and differentiation into effector T cells, and homing functions as a result of interaction with APCs. (III) Diabetogenic effector T cells migrate into the pancreas and infiltrate pancreatic islets causing insulitis and eventually destruction of insulin-producing β-cells.

Despite the daily burden of monitoring blood sugar, taking insulin, and experiencing episodic and occasionally severe hypoglycemia, T1D patients can enjoy a relatively healthy life for decades. Consequently, the safety standards for immunotherapeutic intervention for T1D are high. The risk/benefit balance must clearly exceed that of insulin analog therapy for such interventions to be widely acceptable. The main serious risk associated with most current immunotherapeutic approaches is that of immunosuppression. Avoiding immunosuppression is a major challenge given that diabetogenic T cells in reality are “misguided” effector T cells that direct their destructive power against islet autoantigens instead of foreign pathogens. Major islet autoantigens are derived from insulin, glutamic acid decarboxylase (GAD65), insulinoma-associated antibody 2 (IA-2), and zinc transporter 8 (ZnT8) proteins (Nakayama et al., 2005; Wenzlau et al., 2009). Recognition of these autoantigens, primarily insulin peptide, by diabetogenic T cells initiates the autoimmune response that leads to the ultimate destruction of insulin-producing β-cells and hyperglycemia (**Figure 1)**. In the process, diabetogenic T cells utilize the same recognition systems and costimulatory pathways as do effector T cells specific for invading pathogens. These common systems and pathways regulate T cell proliferation, differentiation, cytokine secretion, and homing to the site of action whether it is an autoimmune target organ or an infected tissue. Therefore, non-specific targeting of a vast array of molecules that cripple islet reactive T cells and prevents autoimmunity can also impair protective T cell function thereby impinging on the host’s ability to mount effective immune responses against invading pathogens. An ideal immunotherapeutic approach would be a one that induces immunologic tolerance to islet autoantigens in high risk individuals and new-onset T1D patients without causing long-term immune suppression. Hypothetically, this can be achieved by developing strategies to selectively eliminate and/or immunoregulate diabetogenic T cells without disrupting normal immune homeostasis or host defense. Despite remarkable achievements in our understanding of basic immunology and disease processes, to date no mechanisms have been identified that selectively inhibit activation of autoreactive T cells without impairing responsiveness of protective T cells. This dilemma has greatly impeded progress in developing successful immunotherapy for T1D.

In the presumed absence of immunologic targets that can be safely targeted to prevent autoimmunity without comprising host defense, some creative approaches have been developed to minimize side effects of targeting molecules by modulating/resetting the immune system through global but transient deletion of T cells or B cells using anti-CD3 or anti-CD20 mAb, respectively. In spite of some early successes in phase I and II clinical trials (Bolt et al., 1993; Herold et al., 2005; van Belle et al., 2011), different anti-CD3 mAbs (Otelixizumab, Teplizumab) failed to meet primary efficacy endpoints in recent phase III clinical trials to impact primary outcomes including hemoglobin A1c (HbA1C), insulin requirements or C-peptide values. A higher dose of the humanized CD3-antibody, ChAglyCD3 (Otelixizumab) led to a decline in insulin requirements after 4 years when compared with placebo treatment. However, the higher dose was associated with significant cytokine release symptoms on infusion days, and led to reactivation of Epstein Barr virus infection (Keymeulen et al., 2010). In the Protégé Study, new-onset T1D subjects who received the highest dose of teplizumab had less decline in C-peptide secretion when compared with placebo, allowing glycemic control to be achieved at a lower insulin dose, indicating a partial therapeutic effect (Sherry et al., 2011). Anti-CD20 antibody is another potential T1D therapy under investigation. CD20 is expressed on the surface of all B cells except plasma cells. Clinical trials with the anti-CD20 mAb Rituximab yielded some degree of success as it helped to preserve residual insulin production in new-onset T1D patients, reduced insulin requirements, and lowered autoantibody levels and HbA1c (Pescovitz et al., 2009; Yu et al., 2011). There were, however, adverse effects including long-term depressed IgM levels, which increases the risk of immunosuppression. Moreover, patients receiving the drug reported a range of side effects including fever, rash, nausea, hypotension, and tachycardia. These studies show that current T cell and B cell antibodies have significant adverse effects, and at best incompletely prevent progressive β-cell loss. They therefore call into question the utility and effectiveness of anti-CD3 or anti-CD20 as stand alone immunotherapies to preserve β-cell function (Sherry et al., 2011; and unpublished data presented at the American Diabetes Association Annual Meeting, San Diego, CA, USA in June 2011).

One promising approach still in its infancy is to utilize Treg cells to selectively inhibit autoreactive T cells. This approach has been challenged by the inability to selectively expand and maintain Treg cells, as reduced frequency or function of Treg cells, or both, is believed to underlie autoimmune diabetes (Atkinson et al., 2011) and other immune-mediated diseases (Koreth et al., 2011; Saadoun et al., 2011). However, recent studies have shown that low doses of IL-2 can be used to promote survival of Treg cells in the non-obese diabetic (NOD) mouse model (Tang et al., 2008) and even to reverse new-onset diabetes (Grinberg-Bleyer et al., 2010). In a clinical trial of IL-2 and Sirolimus in autoimmune diabetes (Proleukin and Rapamune in Type 1 diabetes; ClinicalTrials.gov number, NCT00525889), natural killer-cell count increased and may have been responsible for transient decrease in the function of β-cells. Nonetheless, low dose of IL-2 therapy has been shown to preferentially induce Treg expansion and lead to reversing of immune-mediated human diseases (Koreth et al., 2011; Saadoun et al., 2011), whereas a high dose treatment results in a relative increase in effector T cells population (Bluestone, 2011).

An approach we are advocating is to target the Fas pathway, the prototypical extrinsic death pathway that regulates T cell homeostasis (Nagata and Golstein, 1995). In this perspective, we will discuss the potential of targeting Fas ligand (FasL) as a novel approach to prevent autoimmune destruction of β-cells that is strongly merited by recent findings in the NOD mouse.

## THE Fas PATHWAY: A POTENTIAL THERAPEUTIC TARGET FOR T1D

**FIGURE 2:**
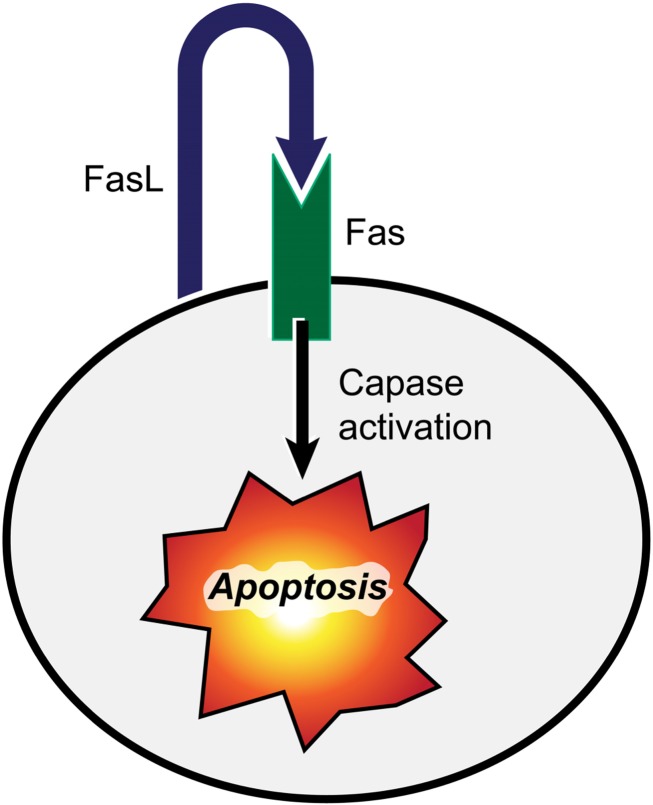
**FasL interaction with its receptor (Fas) triggers cell death.** Binding of FasL to Fas induces the apoptosis of Fas-bearing cells by activation of the caspase signaling cascade leading to cleavage of nuclear DNA and proteolysis of vital cellular proteins.

It has long been known that the Fas pathway plays an important role in maintaining homeostasis of the immune system. Pioneering studies by Nagata and Golstein (1995) have established the Fas pathway as the prototypical extrinsic death pathway. FasL, a tumor necrosis factor-related type II transmembrane protein, initiates an apoptosis signaling cascade by binding to Fas (also known as CD95 or Apo-I) on the target cell triggering cell death (**Figure 2)**. Fas/FasL interaction leads to the formation of death-inducing signaling complex (DISC) that includes Fas-associated protein with death domain (FADD) and aspartate-specific cysteine protease, caspase-8 (Nagata and Golstein, 1995). FADD-mediates activation of the proteolytic activity of caspase 8, which is essential for Fas-induced apoptosis (Denault and Salvesen, 2002). Active caspase-8 leaves the DISC and proteolytically activates downstream effector caspases such as caspase-3 and caspase-7 that perform the bulk of the proteolysis of vital cellular proteins and cleavage of internucleosomal DNA, a hallmark of apoptosis (Lenardo, 1996).

At the cellular level, death of TCR activated hybridomas and primary T cells upon Fas/FasL interactions *in vitro* led to establishment of the paradigm that Fas-mediated activation-induced cell death (AICD) is a major negative regulator of T cell clonal expansion (Brunner et al., 1995; Dhein et al., 1995; Ju et al., 1995). The discovery that T cell lymphoproliferation in lpr and gld mice is due to point mutations in Fas and FasL, respectively, confirmed the physiologic role of the Fas pathway in regulating T cell homeostasis (Nagata and Suda, 1995). Nevertheless, the biological context in which the Fas pathway regulates T cell homeostasis *in vivo* remains unclear. The basis of the unusual composition of T cells that cause lymphoproliferation in mice bearing homozygous lpr or gld mutations is poorly understood. The lymphoproliferation is predominantly caused by a subset of double negative αβ T cells (hereafter referred to as DN T cells) that lack both CD4 and CD8 coreceptors and that is a rare component of the normal T cell population in the secondary lymphoid organs. Thymic negative selection proceeds normally in mutant mice ruling out defective T cell development as a major cause of lymphoproliferation (Kotzin et al., 1988; Singer et al., 1989; Mountz et al., 1990; Zhou et al., 1992). Furthermore, whereas some early studies indicated a delay or defect in deletion of Fas-deficient T cells in response to stimulation by foreign antigens (Gillette-Ferguson and Sidman, 1994; Mogil et al., 1995), recent studies reported minor or no disruption of effector T cell clearance in mice with impaired Fas pathway (Gonzalo et al., 1994; Lohman et al., 1996; Miethke et al., 1996; Hildeman et al., 2002). Consistently, humans and mice with defective Fas pathway efficiently show no defects in clearance of excess effector T cells following acute immune responses (Strasser et al., 2009). Indeed, it is becoming increasingly clear that the proapoptotic molecule Bim (BCL-2 interacting mediator of cell death) is the major regulator of foreign antigen-activated T cell apoptosis *in vivo* (Bouillet and O’Reilly, 2009). Furthermore, because the Fas pathway mainly regulates apoptosis, mice with impaired Fas pathway show no defect in clearing viral infections and remain immunocompetent (Watanabe-Fukunaga et al., 1992; Hildeman et al., 2002; Hamad, 2010). Thus, immune responses to acute infections appear to proceed remarkably normal in the absence of functional Fas pathway. However, deletion of chronically activated T cells due to infections with persistent pathogens appears to be impaired in mutant mice (Stranges et al., 2007; Bouillet and O’Reilly, 2009). In addition, chronic activation by self antigens may be a factor in driving DN T cell accumulation even in germfree (GF) mice, suggesting no major role for microbiota in the process (Maldonado et al., 1999).

Early historic rise and fall of interest in the Fas pathway as an immunomodulator of T1D.

The discovery in the early 1990s of the loss-of-function mutations in Fas (called the lpr mutation) and FasL (called the gld mutation) enabled the assessment of Fas and FasL on the diabetogenic process in the widely used NOD mice (Nagata and Suda, 1995). The initial finding that NOD mice bearing homozygous lpr or gld mutations are completely protected from autoimmune diabetes (Chervonsky et al., 1997; Su et al., 2000; Petrovsky et al., 2002; Mohamood et al., 2007) unveiled the pivotal role for the Fas pathway in driving the pathogenic process of autoimmune diabetes and led to great excitement in the therapeutic potential of targeting the Fas pathway. Based on the physiological role of Fas/FasL interaction in mediating cell death and that TCR activation leads to FasL upregulation, it was presumed that FasL expressed on infiltrating T cells engages Fas on the surface of β-cells leading to their apoptosis (Chervonsky et al., 1997). This hypothesis, however, did not materialize because specific deletion of the Fas gene in β-cells did not spare them from autoimmune destruction (Kim et al., 1999; Apostolou et al., 2003). The dispensable role of Fas-mediated apoptosis in destroying β-cells was disappointing and puzzling at the same time, as it became difficult to fathom an alternative mechanism to explain this potent phenomenon. Thereafter, the view that the protective effect of inactivating the Fas pathway on autoimmune diabetes is an epiphenomenon prevailed. This view is enforced by the fact that mice bearing homozygous gld or lpr mutation develop an age-dependent lymphoproliferation that is predominated by double negative αβ T cells that are rare in normal mice (Watanabe-Fukunaga et al., 1992).

The complete protection from insulitis by the gld and lpr mutations in autoimmune diabetes prone NOD mice occurs even though mutant mice develop age-dependent though benign T cell lymphoproliferation. The absence of insulitis and overt diabetes in the presence of large numbers of activated T cells underlies the potency of the protective mechanism(s). Yet the lymphoproliferation is an obviously unwelcome side effect that is commonly associated with the development of anti-nuclear antibodies and lupus-like condition whose severity depends on the genetic background of the mouse strain (Cohen and Eisenberg, 1991). The lymphoproliferation has also frustrated the efforts to investigate and uncover mechanisms by which inactivation of the Fas pathway prevents autoimmune diabetes. As a consequence, the belief that protection from diabetes is an epiphenomenon related to the distortion of the immune system by expansion of DN T cells passed unchallenged and interest in pursuing the Fas pathway as a therapeutic target faded. However, the protective effect of inactivating the Fas pathway has also been seen in other models of organ-specific autoimmune diseases, including multiple sclerosis, and inflammatory conditions (Waldner et al., 1997; Henriques-Pons and de Oliveira, 2009; Ko et al., 2011). Nevertheless, the question of whether the protective effects and lymphoproliferation are consequential or dissociable side effects of inactivating the Fas pathway has remained unanswered for a long period of time.

**FIGURE 3:**
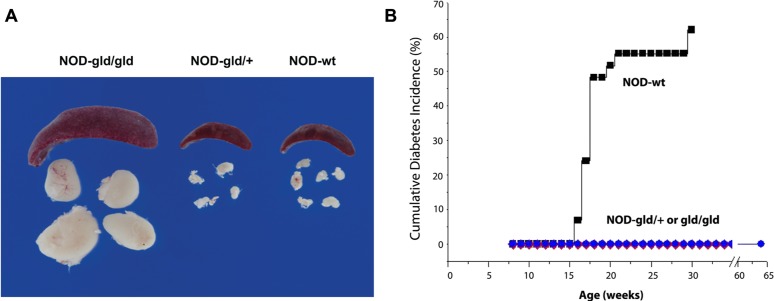
**Single gld allele provides complete protection from diabetes without causing lymphoproliferation.** **(A)** Images of spleens and peripheral lymph nodes of NOD mice bearing homozygous (gld/gld), heterozygous (gld/+) mutation or wt (WT) FasL. **(B)** Homozygous and heterozygous gld mutations completely prevent diabetes in NOD mice as compared to NOD-wt littermates. NOD mice bearing homozygous gld mutations develop an age-dependent lymphoproliferation **(A)** and become completely protected from diabetes **(B)**. NOD mice bearing the heterozygous gld mutation develop no lymphoproliferation but become completely protected from autoimmune diabetes **(B)**. Wild type NOD mice develop no lymphoproliferation but develop autoimmune diabetes (adapted from Mohamood et al., 2007).

**FIGURE 4:**
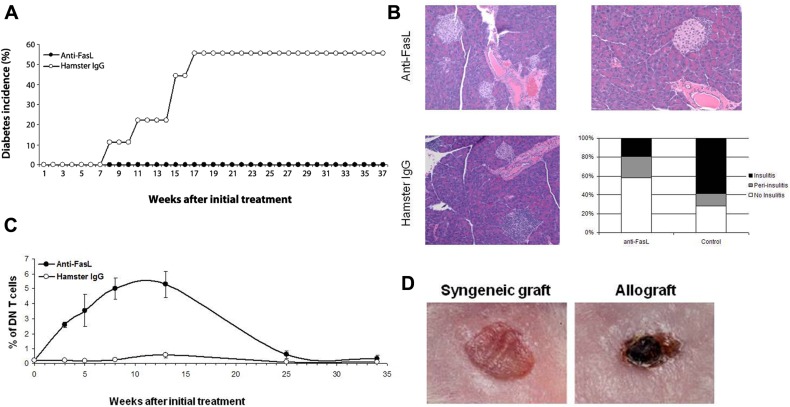
**Anti-FasL treatment protects against diabetes development.** Four-week-old NOD-wt mice were injected weekly, i.p. with 500 μg of anti-FasL MFL4 antibody (*n* = 10) or control hamster IgG (*n* = 9) for two consecutive weeks followed by 300 μg weekly injections until the age of 16 weeks. **(A)** Diabetes incidence in the two groups. **(B)** Anti-FasL treatment curtails insulitis development in NOD-wt mice. Pancreata from three mice in the anti-FasL group or from three non-diabetic mice in the control group were collected 9 weeks after termination of the treatment and analyzed for insulitis. Representative H&E sections show no insulitis (top, left) or peri-insulitis (top, right) in islets of mice that received anti-FasL and severe insulitis in islets of mice in the control group (left, bottom). The histogram shows percent of islets with insulitis (filled bars), peri-insulitis (shaded bars) or no insulitis (open bar) in anti-FasL (120 islets) and control (42 islets) groups. **(C)** Treatment led to an only mild and transient increase in DN T cells. PBL were stained and the frequency of TCR^+^CD4^-^CD8^-^ (DN) cells relative to total T cells was determined. Results are expressed as mean ± SEM. **(D)** Anti-FasL treated NOD-wt mice were grafted with syngeneic skins (left) or allogeneic (right) skin grafts from C3H mice 15 weeks after the last injection. The allograft skins were rejected within 7 days (3/3). Data adapted from Mohamood et al. (2007).

Why revisit the potential of FasL as a therapeutic target in T1D? During the past few years, we have developed encouraging evidence from investigating disease resistance of NOD-gld/+ mice (**Figure 3**) and prevention of diabetes development in NOD-wt mice using a FasL-neutralizing mAb (**Figure 4)**. These studies have shown that the protective effect of targeting FasL is dissociable from the lymphoproliferation: heterozygous gld NOD mice are completely protected from autoimmune diabetes, yet do not develop lymphoproliferation (Su et al., 2000; Nakayama et al., 2002; Mohamood et al., 2007). The translational evidence is the most exciting, as blockade of FasL with MFL4-neutralizing mAb prevents the disease in wild type NOD mice without causing lymphoproliferation (Nakayama et al., 2002; Mohamood et al., 2007). These results indicate that, contrary to the previously long held belief, FasL may be worth dedicated investigation as a therapeutic target. There are unique advantages for targeting FasL that are associated with the following properties of the Fas pathway:

## Fas/FasL INTERACTION IS NOT REQUIRED FOR T CELL ACTIVATION

As postulated by the widely accepted two signals model for T cell stimulation, optimal activation requires one signal to be delivered by the T cell receptor (TCR) engagement of cognate MHC–peptide complex and a second signal provided by the costimulatory molecule CD28 binding to CD80 and CD86 molecules on antigen presenting cells (APCs). Although previous evidence indicated that FasL can costimulate TCR transgenic CD8 T cell proliferation in an adoptive transfer system (Suzuki et al., 2000), the magnitude and potency of normal T cells is largely unaffected and in some circumstances enhanced by the absence of FasL (Hildeman et al., 2002; Mohamood et al., 2008). Therefore, targeting FasL is not expected to negatively impact T cell activation as shown by efficient clearance of acute viral infections by Fas and FasL-deficient mutant mice (Hughes et al., 2008; Hutcheson et al., 2008; Weant et al., 2008).

## Fas/FasL INTERACTION IS NOT REQUIRED FOR DELETION OF EFFECTOR T CELLS GENERATED IN THE ACUTE, NORMAL ADAPTIVE IMMUNE RESPONSE

When T cells respond to acute antigenic stimulation *in vivo* following infection, they become activated, proliferate and differentiate into effector cells that clear the invading pathogen (Marrack and Kappler, 2004). Once the pathogen is cleared, most of the effector T cells are deleted and immune homeostasis is restored. Interestingly, the Fas pathway plays a minor or no role in the deletion of effector T cells generated in response to acute infections. Such effector T cells are normally deleted by the intrinsic death pathway, mainly by the BH3-only Bcl-2 family member Bim (Hildeman et al., 2002). In contrast, the Fas pathway is required for deletion of chronically activated T cells, and most recent evidence from our group indicates that Fas-mediated apoptosis is involved with restricting DNT cells to the epithelial space (Hamad, 2010). Therefore, downmodulating FasL activity is expected to impose little impact on the expansion and contraction of T cells in response to acute infection. In addition, as discussed below, our mouse studies indicate a wide window for downregulating FasL activity to prevent autoimmune diabetes without perturbing immune homeostasis (Su et al., 2000; Mohamood et al., 2007).

## A WIDE THERAPEUTIC WINDOW EXISTS TO DOWN MODULATE FasL ACTIVITY TO PREVENT T1D WITHOUT CAUSING LYMPHOPROLIFERATION

Because FasL functions as a homotrimer that is generated by random pre-association of single chains, expression of one gld allele in NOD hosts (NOD-gld/+) causes FasL haploinsufficiency due to incorporation of at least one gld mutation in about 85% of FasL homotrimers, thereby impairing their ability to bind Fas receptor (Siegel et al., 2000). The effect of FasL on immune homeostasis, the primary physiologic function of the Fas pathway, is negligible as NOD-gld/+ mice maintain normal immune homeostasis with no signs of lymphoproliferation (Mohamood et al., 2007). Nonetheless, as shown in **Figure 3**, FasL haploinsufficiency completely protects NOD-gld/+ mice from developing autoimmune diabetes (Mohamood et al., 2007). Based on these results, we postulate that completely functional FasL is required for driving the autoimmune process whereas only residual FasL function is sufficient for maintaining immune homeostasis (it is estimated that only about 15% of FasL homotrimers are functional in NOD-gld/+ mice yet there are no proliferation or ANA production). The ability of FasL-neutralizing mAb to prevent diabetes development in NOD-wt mice without causing lymphoproliferation or autoantibody production (Su et al., 2000; Nakayama et al., 2002; Mohamood et al., 2007; **Figure 4**) is consistent with the existence of a large functional window to safely downregulate FasL activity for therapeutic purposes. Thus, FasL activity can potentially be targeted to induce organ-specific immunotolerance. This notion may not be limited to autoimmune diabetes as the Fas pathway inactivation also prevents experimental autoimmune encephalomyelitis (EAE), the animal model of multiple sclerosis (Waldner et al., 1997). These new approaches offer breakthrough models to investigate the role of the Fas pathway in autoimmune diabetes that can potentially lead to a novel therapeutic strategy that does not directly target T cells.

## CONCLUSION

Recent data provide proof-of-concept that antibody blockade against FasL can specifically and significantly arrest T1D development *in vivo* (Mohamood et al., 2007; Su et al., 2007). The safety concerns and adverse side effects of antigen non-specific interventions, as well as the lack of permanent remission of disease with any agent tested to date, have heightened interest in identifying non-conventional strategies to modulate the disease. The unique property of FasL as an apoptosis-inducing molecule makes it a potentially attractive target as standalone therapy or a component of complementary immunotherapeutic strategies for autoimmune diseases. Additional research is needed to further investigate this pathway and understanding its specific mechanistic roles in driving development of T1D. It will be particularly interesting to determine whether Fas-mediated apoptosis is mediating cytotoxicity of diabetogenic CD8 T cells, which has recently been implicated in killing β-cells in T1D patients (Bulek et al., 2012; Coppieters et al., 2012) or involved in killing regulatory cells responsible for protecting the pancreas from autoreactive T cells. It is our goal and expectation that this perspective will provoke novel research that will unravel the important, yet complex, role of the Fas signaling pathway in regulating autoimmune diabetes and other organ-specific autoimmune diseases.

## Conflict of Interest Statement

The authors declare that the research was conducted in the absence of any commercial or financial relationships that could be construed as a potential conflict of interest.
